# Insecticide Susceptibility of Field-Collected Populations of *Culex tritaeniorhynchus* in the Republic of Korea

**DOI:** 10.1673/031.013.0201

**Published:** 2013-01-08

**Authors:** Dae-Hyun Yoo, E-Hyun Shin, Dong-Kyu Lee, Young Joon Ahn, Kyu-Sik Chang, Hyun-Kyung Kim, Seong-Yoon Kim, Chan Park

**Affiliations:** 1 Division of Medical Entomology, National Institute of Health, Chungcheongbuk-do 363-951, Republic of Korea; 2 Department of Health & Environment, Kosin University, Busan 606-701, Republic of Korea; 3 WCU Biomodulation Major, Department of Agricultural Biotechnology, Seoul National University, Seoul 151-921, Republic of Korea

**Keywords:** Japanese encephalitis vector, regional variation

## Abstract

The toxicities of 10 insecticides were examined against late third instar *Culex tritaeniorhynchus* Giles (Diptera: Culicidae) using the direct-contact mortality bioassay. Six geospatially-distant field mosquitoes were collected from Chuncheon-si, Hwaseong, Seosan. Jeonju, Daegu, and Busan in the Republic of Korea. Marked regional variations of insecticide susceptibility were observed. Field populations of Seosan, Jeonju, and Daegu from agricultural areas showed higher to extremely higher insecticide susceptibility to pyrethroids than those of Chuncheon-si, Hwaseong, and Busan strains from non-agricultural areas. Extremely high to low levels of susceptibility were measured: bifenthrin, susceptible ratio (SR) = 2.7–896.3; β-cyfluthrin, SR = 1.8–633.3; α-cypermethrin, SR = 1.2–1,051.9; deltamethrin, SR = 1.3–711.1; permethrin, SR = 1.5–1,053.4; etofenprox, SR = 2.2–29.3; chlorfenapyr, SR = 5.1–103.6; chlorpyrifos, SR = 2.3– 337.0; fenitrothion, SR = 2.0–142.3; and fenthion, SR = 1.4–186.2. *Cx. tritaeniorhynchus* populations from rice paddies had been under heavy selection pressure due to the agricultural insecticides, and that's why the mosquito species demonstrated high resistance to pyrethroids, which were used for a long time to control agricultural pests in the localities. These results indicate that careful selection and rotational use of these insecticides may result in continued satisfactory control against field populations of Japanese encephalitis vector mosquitoes.

## Introduction

Japanese encephalitis (JE) is a vector-borne viral disease that occurs in South Asia, Southeast Asia, the Pacific, and East Asia, including the Republic of Korea (ROK) (Solomon 2006). As late as 1982, 1197 cases of JE were reported in the ROK, when the vaccination rate was 45.7% (Sohn 2000). However, government-mandated vaccination and vector-control programs decreased JE in the ROK to 6–7 cases reported annually over the last two decades, except for the recent outbreak of 26 cases in 2010 (Center for Disease Control and Prevention/Korea 2012). *Culex tritaeniorhynchus* Giles (Diptera: Culicidae) is the major vector of JE in the ROK (Burke and Leake 1988; Vaughn and Hoke 1992; Khan et al. 1996). Because *Cx. tritaeniorhynchus* breeds mainly in paddy fields, it is under heavy selection pressure with agricultural applications of insecticides and other pesticides sprayed on the rice fields. This selection pressure has resulted in the development of insecticide resistance (Shim et al. 1995a; Karunaratne and Hemingway 2000; Erlanger et al. 2009; Shin et al. 2011). Agricultural insecticide treatment can exert a selective pressure on both the larval and adult stages of vectors. Some of the breeding sites created by agricultural practices in rice fields and by irrigation schemes are sprayed directly. When agricultural breeding sites are treated, all the mosquito larvae are subjected to selective pressure, which is more likely to induce resistance than when houses are sprayed for mosquito control. Insecticides sprayed in crop fields can be carried by wind to nearby mosquito breeding sites. Finally, rains can wash pesticides applied on crops and drain them into ground pools or ditches where mosquito larvae breed. Adult mosquitoes have been observed resting on treated rice. In the ROK, rice-field-collected mosquito populations have developed locally unique insecticide resistances according to the long-term insecticides used in the region (Shim et al. 1982; Shim et al. 1995b; Chang et al. 2009; Shin et al. 2011).

In the ROK, the local public health centers have controlled mosquitoes, but they have not considered the insecticide resistance of vector mosquitoes and have used various insecticides. As vector mosquitoes obtain insecticide resistance, more insecticides may be applied, which can cause human and environmental health problems. The widespread use of commonly used less expensive insecticides has been a major obstacle in implementing cost-effective and safe integrated programs for mosquito management. In the ROK, 26 cases of JE occurred in 2010 (Center for Disease Control and Prevention/Korea 2012), but little monitoring of local insecticide resistance of *Cx. tritaeniorhynchus* has been performed for integrated vector mosquito control in the last 5 years.

Here, the resistance patterns of 10 currently used insecticides against six geospatially-distant field-collected populations of *Cx. tritaeniorhynchus* are reported. To estimate insecticide resistance of *Cx. tritaeniorhynchus* to agricultural pesticides, the resistance levels of *Cx. tritaeniorhynchus* collected from rice-field and non-rice-field areas were tested and compared.

## Materials and Methods

### Chemicals

The following ten insecticides were purchased from Sigma-Aldrich (http://www.sigmaaldrich.com/) and were used in this study: bifenthrin (97.0% purity), β-cyfluthrin (98.0%), α-cypermethrin (97.5%), deltamethrin (99.5%), etofenprox (96.5%), permethrin (95.5%), chlorpyrifos (98.5%), fenthion (95.5%), fenitrothion (98.5%), and chlorfenapyr (99.0%). Triton X-100 was obtained from Shinyo Pure Chemicals (Osaka, Japan). All other chemicals used were analytical grade and available commercially.

### Mosquitoes

**Figure 1.**
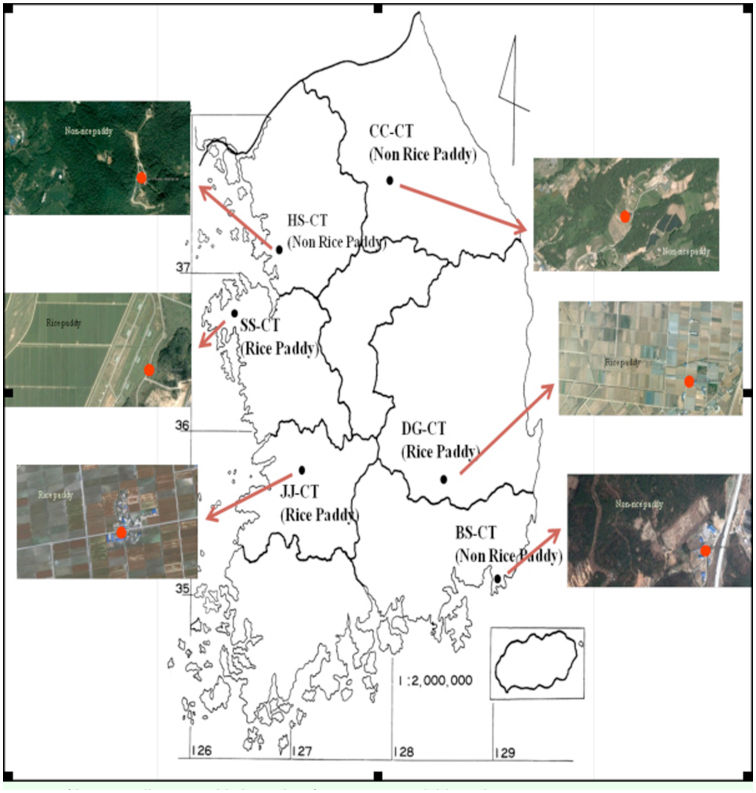
Locations of larvae collections. High quality figures are available online.

Six different colonies of *Cx. tritaeniorhynchus* were established from larvae collected near rice paddy fields and cow sheds in Chuncheon (designated CC-CT; 37° 52′ 56.19″ N, 127° 46′ 12.16″ E), Hwaseong (HS-CT; 37° 12′ 18.40″ N, 126° 50′ 53.19″ E), Busan (BS-CT; 35° 12′ 21.73″ N, 129° 12′ 5.31″ E), Seosan (SS-CT; 36° 42′ 27.49″ N, 126° 28′ 23.97″ E), Jeonju (JJ-CT; 35° 54′ 54.63″ N, 127° 0′ 31.23″ E), and Daegu (DG-CT; 36° 8′ 47.78″ N, 128° 21′ 57.27″ E) from early August to mid-September 2011 (Figure 1). Field strains of CC-CT and HS-CT were collected from small swamps near hog barns, which were surrounded by mountains and at least 3km from a farming village. The BS-CT collection site was an area of small swamps near a hog barn along an urban area, and was surrounded by factories. Field strains of SS-CT, JJ-CT, and DG-CT were collected from small swamps near hog barns, which were surrounded by rice paddies and far from villages. The collected larvae were transferred to an insect rearing room at the Korean National Institute of Health Larvae were reared in plastic trays (27 × 15 × 4 cm) containing 0.5 g of sterilized diet (Vivid S:Super Terramin, 4:1 by weight) (Sewhapet, http://sewhapet.en.ec21.com/). Adult mosquitoes were maintained on a 10% sucrose solution, and were allowed to blood-feed on mice under an approved animal use protocol. All cages were maintained at 27 ± 1° C, with 65–75% relative humidity (RH) and a 12:12L:D photoperiod.

### Bioassay

A direct-contact mortality bioassay (World Health Organization 1981) was used to evaluate the toxicity of the 10 insecticides to late third instar *Cx. tritaeniorhynchus* from each of the six field-collected colonies. Each larvicide was dissolved in methanol, then further diluted in distilled water containing Triton X-100 (20 µl/L). A total of 25 larvae from each colony were placed in paper cups (350 mL) that contained each of the test larvicide solutions (250 mL). The toxicity of each test larvicide was determined using four to six concentrations ranging from 1 to 200 ppm. The control consisted of the methanol-Triton X-100 carrier solution in distilled water.

Treated and control larvae were held under the same conditions used for colony maintenance. Larvae were considered to be dead if they did not move when they were prodded with a fine wooden dowel 24 hrs post-treatment (Perumalsamy et al. 2009). All treatments were replicated three times using 25 larvae/replicate. Because all bioassays could not be conducted at the same time, treatments were blocked over time with a separate control treatment included for each block. Freshly prepared solutions were used for each block of bioassays (Robertson and Preisler 1992).

### Data analysis

Concentration-mortality data were subjected to probit analysis (SAS Institute 2004). The LC_50_ values for each treatment were considered to be significantly different from one another when their 95% confidence limits failed to overlap. The susceptibility ratio (SR) was defined as the LC_50_ value of each field strain divided by the LC_50_ value of the field strain that demonstrated the highest susceptibility to the same insecticide. SR values of < 10, 10–40, 40–160, and > 160 were classified as low, moderate, high, and extremely high resistance, respectively (Kim et al. 2004).

## Results and Discussion

The relative toxicities of 10 insecticides were assayed against third instars of the CC-CT strain and were compared using direct-contact mortality bioassay (Table 1). Based on 24 hr LC_50_ values, chlorpyrifos was the most toxic insecticide, followed by fenitrothion and fenthion. Etofenprox had the lowest toxicity. All insecticide showed low to moderate SR values for the CC-CT strain (SR = 1.0–16.5).

The toxicities of 10 test insecticides were assessed against third instars of the HS-CT strain (Table 2). Based on 24 hr LC_50_ values, the mosquito strain exhibited the highest susceptibility to chlorpyrifos, followed by chlorfenapyr and α-cypermethrin. The HS-CT strain demonstrated low to moderate levels of SR values to all tested insecticides (SR = 1.0– 14.5).

The toxicities of 10 insecticides were evaluated for the BS-CT strain (Table 3). Based on 24 hr LC_50_ values, the BS-CT strain was most susceptible to fenitrothion, followed by fenthion and α-cypermethrin. The larvae showed low to moderate SR values to all tested insecticides (SR = 1.0–15.5).

The SS-CT strain had the highest susceptibility to chlorpyrifos, followed by fenitrothion and chlorfenapyr. The mosquito strain demonstrated the lowest susceptibility to permethrin. The SS-CT strain demonstrated high to extremely high SR values to all tested insecticides (SR = 103.6–1, 053.4), except for etofenprox (SR = 29.3) (Table 4).

**Table 1.**
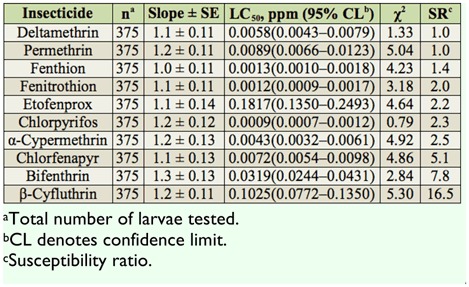
Summary of toxicities of 10 insecticides to third instars of the CC-CT strain of *Culex tritaeniorhynchus* based on the 24 hr exposure contact mortality bioassays.

**Table 2.**
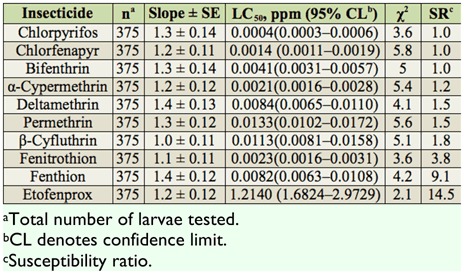
Summary of toxicities of 10 insecticides to third instars of the HS-CT strain of *Culex tritaeniorhynchus* based on the 24 hr exposure contact mortality bioassays.

**Table 3.**
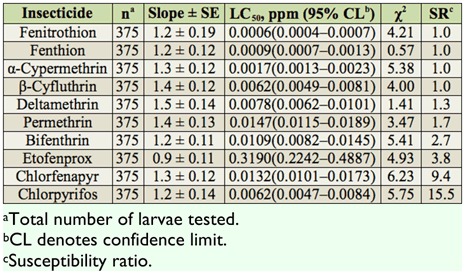
Summary of toxicities of 10 insecticides to third instars of the BS-CT strain of *Culex tritaeniorhynchus* based on the 24 hr exposure contact mortality bioassays.

The JJ-CT strain was highly susceptible to bifenthrin, followed by chlorfenapyr and fenthion. The susceptibility of the JJ-CT strain to permethrin was the lowest. The JJ-CT strain showed a low level of SR value to etofenprox (SR = 1.0), a high level of SR value to chlorfenapyr, fenitrothion, and fenthion (SR = 46.0–80.2), and an extremely high level of SR values (SR = 337.0–958.0) (Table 5).

The DG-CT strain was the most susceptible to chlorpyrifos, followed by chlorfenapyr and fenitrothion (Table 6). The DG-CT strain showed a low level SR value to chlorfenapyr (SR = 6.2), a moderate level SR value to etofenprox, chlorpyrifos, and fenitrothion (SR = 21.8–29.8), a high level SR value to fenthion (SR = 65.4), and an extremely high level of SR values to bifenthrin, β-cyfluthrin, deltamethrin, α-cypermethrin, and permethrin (SR = 236.2–838.2).

Because *Cx. tritaeniorhynchus* breeds mainly in paddy fields, it is under heavy selection pressure due to the agricultural applications of insecticides (Watanabe et al. 1990; Kamimura and Arakawa 1991; Shim et al. 1995a; Karunaratne and Hemingway 2000). The average insecticide susceptibilities of mosquito strains were compared between two different ecological conditions of habitat, namely rice paddy fields (agricultural areas, CC-CT, HS-CT, and BS-CT strains) and non rice paddy fields (non-agricultural areas, SS-CT, JJ-CT, and DG-CT strains) (Table 7). Three *Cx. tritaeniorhynchus* strains from agricultural areas had significantly lower susceptibility to all of the insecticides tested than mosquito strains from non-agricultural areas (average SR values = 28.5–686.3), except for the chemicals etofenprox, chlorfenapyr, and chlorpyrifos. Antonio-Nkondjio et al. (2011) showed that urban pollution had little effect on the devolpment of insecticide resistance in *An. gambiae.* The low SR value of etofenprox might have resulted from its low toxicity to mosquitoes. Of the 10 tested insecticides, etofenprox had the lowest toxicity for a susceptible strain of *Cx. tritaeniorhynchus,* and it had a low SR value when relative levels of resistance of fieldcollected *Cx. tritaeniorhynchus* larvae during 2010 were compared to those of a colony established in 1992 from the same locality (Shin et al. 2011).

**Table 4.**
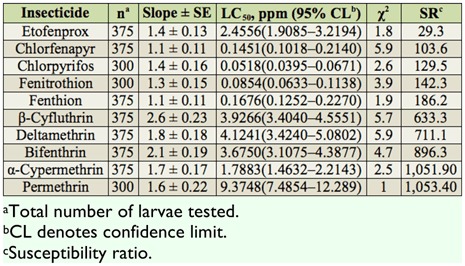
Summary of toxicities of 10 insecticides to third instars of the SS-CT strain of *Culex tritaeniorhynchus* based on the 24 hr exposure contact mortality bioassays.

**Table 5.**
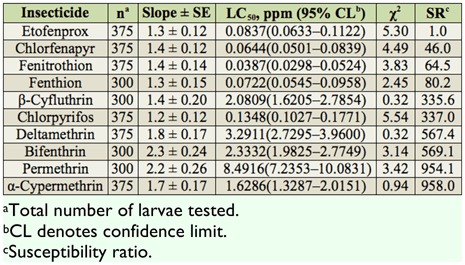
Summary of toxicities of 10 insecticides to third instars of the JJ-CT strain of *Culex tritaeniorhynchus* based on the 24 hr exposure contact mortality bioassays.

**Table 6.**
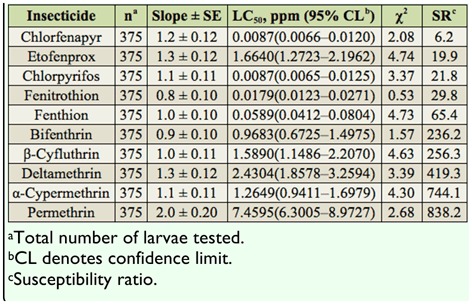
Summary of toxicities of 10 insecticides to third instars of the DG-CT strain of *Culex tritaeniorhynchus* based on the 24 hr exposure contact mortality bioassays.

**Table 7.**
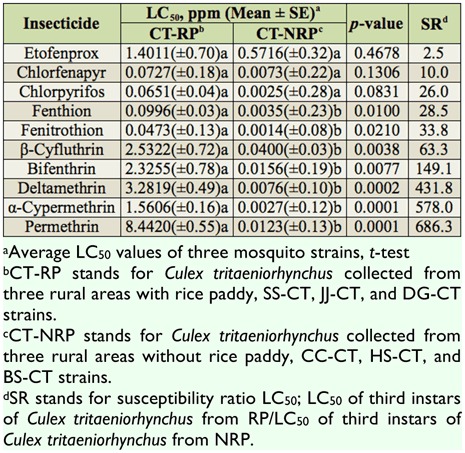
Comparisons of average susceptibilities of 10 insecticides to third instars of field-collected *Culex tritaeniorhynchus* between two different ecological conditions of habitat based on the 24 hr exposure contact mortality bioassays.

Pyrethroid insecticides have been employed frequently in the ROK due to their strong insecticidal activity and relatively lower human toxicity when compared to organophosphate insecticides (Chang et al. 2009). As a result of their increased usage, they demonstrated high to extremely high levels of SR values, while the less used organophosphates demonstrated low to moderate SR values. In the monitoring areas of this study, pyrethroid insecticides have been used for the control of agricultural pests for a long period, and this extended use may be why *Cx. tritaeniorhynchus* demonstrated high resistance to pyrethroids. According to the pest control operators in the area, mosquito control with permethrin failed some years (unpublished data.2011).

The mechanisms of pyrethroid resistance in mosquitoes can be explained by metabolic detoxification (Liu et al. 2006). The metabolic enzymes involved include cytochrome P450s, glutathione transferases, and esterases or carboxylesterases, which hav effects on the detoxification and metabolism of compounds like the pyrethroids (Scott 1991; Feyereisen 1999; Scott 1999). The metabolic detoxification in the pyrethroid resistance of mosquitoes can be decreased using piperonyl butoxide , S,S,S-tributylphosphorotrithioate, and diethyl maleate, which are inhibitors of cytochrome P450 monooxygenases, hydrolases, and glutathione S-transferases, respectively. The mosquitoes pretreated with piperonyl butoxide demonstrated a decreased P450 monooxygenase-mediated detoxification to two permethrin resistance strains of *Culex quinquefasciatus* (Xu et al. 2005). Xu (2005) also reported S, S, S-tributylphosphorotrithioate and diethyl maleate increased the toxicity of permethrin by the weakening of hydrolase-and glutathione S-transferases-mediated metabolic detoxification. However, the authors suggested that these metabolic enzymes did not completely abolish resistance to permethrin, because one or more additional resistance mechanisms involved in overall resistance were largely unaffected by these synergists (Liu et al. 2004). The pyrethroid resistance of *Culex pipiens pipiens* was almost completely suppressed using piperonyl butoxide, reducing cytochrome P450- and carboxylesterase-mediated detoxification in resistance (McAbee et al. 2004). Although further studies on mode of actions between the metabolic enzymes and the synergists are needed, an application of the piperonyl butoxide, S,S,S-tributylphosphorotrithioate, and diethyl maleate to the areas in this study might be very useful for the control of pyrethroid resistance *Cx. tritaeniorhynchus.*

Resistance monitoring is an effective component in a resistance management approach because it provides current information on the responses of *Cx. tritaeniorhynchus* populations to insecticides.

Susceptibility tests need to be conducted over a broad area, as insecticide pressures and usage may vary geographically. Insecticide failures in the ROK have occurred most likely as a result of the development of resistance (Shim et al. 1995a, b; Kim et al. 2007; Chang et al. 2009). Early detection of trends in the development of potential resistance can facilitate the use of synergists, the rotation of insecticides and/or classes of insecticides, or the use of alternate technologies that reduce the usage of chemical insecticides (Yilma et al. 1991; Lee et al. 1997). A better understanding of the mechanisms governing resistance development in *Cx. tritaeniorhynchus* will be extremely important for developing novel strategies to circumvent and/or delay resistance development, controlling resistant mosquitoes and, ultimately, reducing the prevalence of mosquito-borne diseases like JE in the ROK.

## Abbreviations

**BS-CT**,: Busan
**CC-CT**,: Chuncheon
**DG-CT**,: Daegu
**HS-CT**,: Hwaseong
**JE**,: Japanese encephalitis
**JJ-CT**,: Jeonju
**ROK**,: Republic of Korea
**SR**,: susceptibility ratio
**SS-CT**,: Seosan
